# Spray Fluorescent Probes for Fluorescence-Guided Neurosurgery

**DOI:** 10.3389/fonc.2019.00727

**Published:** 2019-08-06

**Authors:** Yosuke Kitagawa, Shota Tanaka, Yugo Kuriki, Kyoko Yamamoto, Akira Ogasawara, Takahide Nejo, Reiko Matsuura, Tsukasa Koike, Taijun Hana, Satoshi Takahashi, Masashi Nomura, Shunsaku Takayanagi, Akitake Mukasa, Mako Kamiya, Yasuteru Urano, Nobuhito Saito

**Affiliations:** ^1^Department of Neurosurgery, Graduate School of Medicine, The University of Tokyo, Tokyo, Japan; ^2^Laboratory of Chemistry and Biology, Graduate School of Pharmaceutical Sciences, The University of Tokyo, Tokyo, Japan; ^3^Laboratory of Chemical Biology and Molecular Imaging, Graduate School of Medicine, The University of Tokyo, Tokyo, Japan; ^4^Genome Science Division, Research Center for Advanced Science and Technology, The University of Tokyo, Tokyo, Japan; ^5^Department of Neurosurgery, Graduate School of Medical Sciences, Kumamoto University, Kumamoto, Japan

**Keywords:** fluorescent probe, glioma surgery, hydroxymethyl rhodamine green, topical, 5-aminolevulinic acid

Surgery is the initial and most important mode of treatment for glioma, the most common primary brain tumor. The greatest possible extent of resection and preservation of neurological functions are both the primary goals, although they tend to be in the trade-off relationship. Several surgical adjuncts have been developed for maximal safe resection, which include electrophysiological monitoring, navigation system, intraoperative imaging, and fluorescent probes. Intraoperative imaging such as MRI and ultrasound tend to overcome inacuracy due to brain shift during tumor resection. However, these devices tend to be costly. In contrast, fluorescent probes can be implemented to surgery by simply attaching an excitation and emission filters to a preexisting microscope, achieving high versatility with low cost. Fluorescence-guided surgery has been proven effective for radical tumor resection; fluorescent probes effectively visualize the tumor intraoperatively.

Fluorescence probes are useful indicators for biologically relevant targets, being sensitive, rapidly responsive, and capable of affording high spatial resolution via microscopic imaging without destroying structures (1). In surgery, many different types of fluorescent probes have been investigated in preclinical and clinical studies. They are classified based on the mechanisms of their actions into “always-on” probes and activatable probes (2). “Always-on” probes, administered with intravenous injection, readily reveal hypervascularized areas, differentiating the tumor from the normal brain parenchyma. These probes have several disadvantages in their use. They do not necessarily accumulate selectively in tumor tissues (3). Even if they do, they show high background signals, which makes it difficult to identify tumor tissues. On the contrary, activatable probes are efficient in identifying the tumor specifically either with its high substance accumulation or with its metabolic activation, although they often require a considerable time up to several hours in clinical use (4). One such example is activatable cell-penetrating peptide (ACPP) (5). The ACPP probe is a fluorescently labeled, polycationic cell-penetrating peptide coupled with a cleavable linker. When the probe is exposed to a protease being active in the tumor, the linker is cleaved and the inhibitory peptide is dissociated. Then it binds to and enters the tumor cells.

Three types of fluorescent probes are in use in neurosurgical procedures: 5-aminolevulinic acid (5-ALA), indocyanine green (ICG), and fluorescein sodium (6). ICG and fluorescein sodium are classified as “always-on” probes (7), whereas 5-ALA is classified as an activatable probe. It induces the production of protoporphyrin IX (PpIX), which preferentially accumulates in glioma tissues. It reaches its peak 6 h after 5-ALA administration (8).

A randomized controlled study conducted by Stummer et al. assessed the efficacy of fluorescence-guided resection with 5-ALA in patients with malignant gliomas amenable to complete resection of contrast-enhancing tumor (9). The primary endpoints were the number of patients without contrast-enhancing tumor in early postoperative MRI and 6-month progression-free survival. Secondary endpoints were volume of residual tumor on postoperative MRI, overall survival, neurological deficit, and toxic effects. Patients assigned to fluorescence-guided surgery had a higher 6-month progression-free survival than did those assigned to conventional surgery (41.0 vs. 21.1%, *p* = 0.0003). The complete resection rate of contrast enhancing tumor in the fluorescence-guided surgery group compared favorably with that in the conventional surgery group (65 vs. 36%, *p* < 0.0001). They have shown that the use of 5-ALA resulted in a higher complete resection rate and a longer progression-free survival. Of note, no significant differences in overall survival, neurological deficit, or toxic effects were noted between the two groups. 5-ALA is now routinely used in clinical practice for high-grade glioma surgery. On the contrary, it fails to identify low-grade gliomas. There are some limitations in its use as well, which would include false positivity, false negativity, the need for preoperative oral administration, and the inability for re-administration (10).

We have previously reported on hydroxymethyl rhodamine green (HMRG) probes as activatable fluorescent probes for rapid cancer detection with topical spray (11). They are comprised of HMRG as fluorescent scaffold combined with various types of amino acids or dipeptides. Their fluorescence are completely quenched by spirocyclic caging but are activated rapidly with a one-step enzymatic reaction in the presence of specific aminopeptidase enzymes within a few to tens of minutes (Figure 1). Dipeptidylpeptidase IV and γ-glutamyltransferase (GGT) are some examples of dipeptidyl peptidases and aminopeptidases that are known to be expressed at the elevated levels in various types of cancers, such as hepatic cancer, esophagus cancer, ovarian cancer, and glioma as well, as compared to normal tissues (11–15). GGT is present on the plasma membrane and catalyzes the transfer of the N-terminal gamma-glutamyl group of substrates to acceptor molecules. When it encounters GGT on the surface of a cancer tissue, it is hydrolyzed by the enzymatic reaction to yield the highly fluorescent product, HMRG. *In vitro*, when red fluorescent protein was used as a reference for location of a cell of the ovarian cancer cell line SHIN3, sensitivity and specificity of detecting SHIN3-RFP tumors with gGlu-HMRG 10 min after injection were 100 and 100%, respectively. *In vivo*, after establishing the intraperitoneal dissemination mouse model using SHIN3, minute tumors <1 mm in diameter in the peritoneum could be readily identified as early as 30 s after spraying the gGlu-HMRG probe under fluorescence-guided endoscopy and they could be removed easily with forceps. Without fluorescent probes, confirming the completeness of resection would be difficult with naked eyes, and therefore would have to be ascertained by postoperative histological confirmation (16–18).

**Figure 1:**
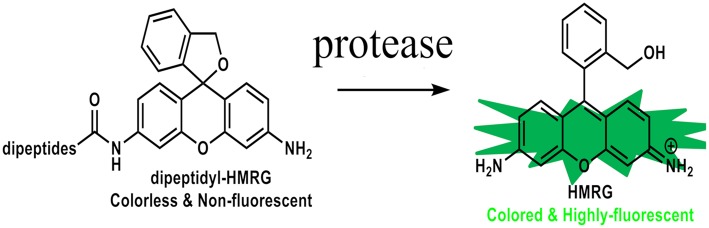
Chemical structures of HMRG-based fluorescent probes. These probes are completely quenched due to spirocyclic caging but are activated with protease reaction.

These HMRG probes can be applied for the detection of a brain tumor. Some probes fluorescence glioma tissue more than the surrounding normal brain tissue after their topical application (Figure 2). The biggest advantages of these activatable probes would be that they start to react immediately after spray and can be administered repeatedly during surgery with potentially high specificity and sensitivity. Topical application of spray probes can be performed with a much lower dose than systemic administration, and thus they are deemed safer. On the other hand, one caveat would be that the efficacy of spray fluorescent probes may be diminished by being washed away by active bleeding at the resection cavity before they provoke fluorescence in the tumor. Another concern would be that the surrounding brain tissue may overhang the resection cavity, which makes it difficult to efficiently apply fluorescent probes to the cavity and to accurately evaluate the degree of fluorescence.

**Figure 2:**
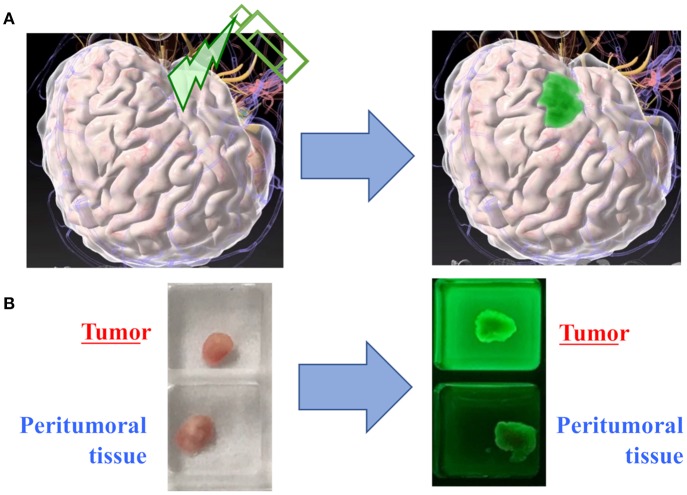
Proof of concept in spray fluorescent probes. **(A)** The conceptual image of tumor detection with topical application of a fluorescent probe. **(B)** In a representative case of glioma, the fresh tumor tissue exhibits significantly stronger fluorescence as compared to the peritumoral tissue.

We believe that these novel fluorescent probes have a potential as probes complementary to 5-ALA in high-grade glioma surgery. As discussed earlier, 5-ALA is highly effective and routinely administered for resection of high-grade gliomas, but these probes would be worth a try when the tumor does not fluorescence with 5-ALA or when 5-ALA fluorescence has significantly diminished at the end of tumor resection. In addition, they would be a breakthrough in low-grade glioma surgery, where 5-ALA is largely ineffective. Moreover, they are potentially applicable to virtually all types of brain tumors including malignant lymphoma and brain metastasis, which are the top differential diagnoses of glioma. Novel HMRG probes which specifically detect malignant lymphoma or brain metastasis are currently under investigation. Last but not the least, a larger clinical study is warranted to assess the clinical utility of our probes in brain tumor surgery.

## Author Contributions

YKi, ShoT, MK, and YU wrote the manuscript. YKu, KY, AO, TN, RM, TK, TH, SatT, MN, ShuT, AM, and NS carefully reviewed the manuscript.

### Conflict of Interest Statement

The authors declare that the research was conducted in the absence of any commercial or financial relationships that could be construed as a potential conflict of interest.

## References

[1] Tsien RY Fluorescent and photochemical probes of dynamic biochemical signals inside living cells. In: Czarnik AW, editor. American Chemical Society. Washington, DC (1993). p. 130–46.

[2] Kobayashi H Ogawa M Alford R Choyke PL Urano Y. New strategies for flourescent probe desogn in medical diagmpstic imaging. Chem Rev. (2011) 110:2620–40. 10.1021/cr900263j 20000749 PMC3241938

[3] Diaz RJ Dios RR Hattab EM Burrell K Rakopoulos P Sabha N. Study of the biodistribution of fluorescein in glioma-infiltrated mouse brain and histopathological correlation of intraoperative findings in high-grade gliomas resected under fluorescein fluorescence guidance. J Neurosurg. (2015) 122:1360–9. 10.3171/2015.2.JNS132507 25839919

[4] Kobayashi H Choyke PL. Target-cancer-cell-specific activatable fluorescence imaging probes: rational design and *in vivo* applications. Acc Chem Res. (2011) 44:83–90. 10.1021/ar1000633 21062101 PMC3040277

[5] Nguyen QT Olson ES Aguilera TA Jiang T Scadeng M Ellies LG. Surgery with molecular fluorescence imaging using activatable cell-penetrating peptides decreases residual cancer and improves survival. Proc Natl Acad Sci USA. (2010) 107:4317–22. 10.1073/pnas.0910261107 20160097 PMC2840114

[6] Belykh E Martirosyan NL Yagmurlu K Miller EJ Eschbacher JM Izadyyazdanabadi M. Intraoperative fluorescence imaging for personalized brain tumor resection: current state and future directions. Front Surg. (2016) 3:55. 10.3389/fsurg.2016.00055 27800481 PMC5066076

[7] Eyupoglu IY Hore N Fan Z Buslei R Merkel A Buchfelder M. Intraoperative vascular DIVA surgery reveals angiogenic hotspots in tumor zones of malignant gliomas. Sci Rep. (2015) 5:1–7. 10.1038/srep07958 25609379 PMC4302292

[8] Stummer W Stepp H Moller G Ehrhardt A Leonhard M Reulen HJ. Technical principles for protoporphyrin-ix-fluorescence guided microsurgical resection of malignant glioma tissue. Acta Neurochir. (1998) 140:995–1000. 10.1007/978-3-319-93698-7_49 9856241

[9] Stummer W Pichlmeier U Meinel T Wiestler OD Zanella F Reulen HJ. Fluorescence-guided surgery with 5-aminolevulinic acid for resection of malignant glioma: a randomised controlled multicentre phase III trial. Lancet Oncol. (2006) 7:392–401. 10.1016/S1470-2045(06)70665-9 16648043

[10] Ferraro N Barbarite E Albert TR Berchmans E Shah AH Bregy A. The role of 5-aminolevulinic acid in brain tumor surgery: a systematic review. Neurosurg Rev. (2016) 39:545–55. 10.1007/s10143-015-0695-2 26815631

[11] Urano Y Sakabe M Kosaka N Ogawa M Mitsunaga M Asanuma D. Rapid cancer detection by topically spraying a γ-glutamyltranspeptidase-activated fluorescent probe. Sci Transl Med. (2011) 3:110ra119. 10.1126/scitranslmed.3002823 22116934 PMC7451965

[12] Stremenova J Krepela E Mares V Trim J Dbaly V Marek J. Expression and enzymatic activity of dipeptidyl peptidase-IV in human astrocytic tumours are associated with tumour grade. Int J Oncol. (2007) 31:785–92. 10.3892/ijo.31.4.785 17786309

[13] Onoyama H Kamiya M Kuriki Y Komatsu T Abe H Tsuji Y. Rapid and sensitive detection of early esophageal squamous cell carcinoma with fluorescence probe targeting dipeptidylpeptidase IV. Sci Rep. (2016) 6:1–7. 10.1038/srep26399 27245876 PMC4887889

[14] Fujii T Kamiya M Urano Y. *In vivo* imaging of intraperitoneally disseminated tumors in model mice by using activatable fluorescent small-molecular probes for activity of cathepsins. Bioconjug Chem. (2014) 25:1838–46. 10.1021/bc5003289 25196809

[15] Liu Y Tan J Zhang Y Zhuang J Ge M Shi B. Visualizing glioma margins by real-time tracking of γ-glutamyltranspeptidase activity. Biomaterials. (2018) 173:1–10. 10.1016/j.biomaterials.2018.04.053 29727797

[16] Braun S Vogl FD Naume B Janni W Osborne MP Coombes RC. A pooled analysis of bone marrow micrometastasis in breast cancer. N Engl J Med. (2005) 353:793–802. 10.1056/NEJMoa050434 16120859

[17] Cote RJ Rosen PP Lesser ML Old LJ Osborne MP. Prediction of early relapse in patients with operable breast cancer by detection of occult bone marrow micrometastases. J Clin Oncol. (1991) 9:1749–56. 10.1200/JCO.1991.9.10.1749 1919627

[18] Lugo TG Braun S Cote RJ Pantel K Rusch V. Detection and measurement of occult disease for the prognosis of solid tumors. J Clin Oncol. (2003) 21:2609–15. 10.1200/JCO.2003.01.153 12829682

